# Magnitude of anemia and associated factors among pregnant women attending antenatal care in governmental health facilities of Shashemene Town, Oromia region, Ethiopia

**DOI:** 10.3389/fpubh.2024.1409752

**Published:** 2024-09-04

**Authors:** Mekiya Nasir, Habtamu Molla Ayele, Rameto Aman, Kelil Hussein

**Affiliations:** ^1^Department of Public Health, School of Health Science, Madda Walabu University, Bale Robe, Ethiopia; ^2^Maternal and Child Health Directorate, Federal Ministry of Health, Addis Ababa, Ethiopia

**Keywords:** anemia, pregnant women, hemoglobin, Shashemene, Ethiopia

## Abstract

**Introduction:**

Anemia during pregnancy is a common issue that significantly affects the health of both the mother and her child. Globally, anemia is a major public health concern, affecting both developing and developed countries, with approximately 1.3 billion people affected. Pregnant women are among the most vulnerable to anemia.

**Objective:**

To assess the magnitude and risk factors of anemia among pregnant women attending antenatal care in Shashemene Town, Oromia, Ethiopia.

**Methods:**

A facility-based cross-sectional study was conducted among 391 pregnant women in Shashemene Town in April 2022. Data were collected using interviewer-administered questionnaires, along with laboratory examinations of blood and stool samples. The data were entered into EpiData 3.1 and analyzed using the Statistical Package for Social Sciences (SPSS) version 22. Bivariate logistic regression was performed, and variables with a *p*-value of <0.25 were included in the multivariate logistic regression analysis to identify factors associated with anemia. Adjusted odds ratio (AOR) with 95% CIs were calculated, and a *p*-value of < 0.05 was considered statistically significant. Finally, the results are presented using narration, descriptive statistics, such as tables, graphs, and charts.

**Results:**

The prevalence of anemia was found to be 30.9% (95% CI: 26.4, 35.4%). Factors significantly associated with a reduced risk of anemia included high dietary diversity (AOR = 0.217, 95% CI: 0.105–0.451), no history of excessive menstrual bleeding (AOR = 0.162, 95% CI 0.076–0.345), age 25–34 years (AOR = 0.391, 95% CI 0.173–0.883), and age ≥ 35 years (AOR = 0.068, 95% CI 0.011–0.444). Conversely, a mild upper arm circumference (MUAC) of <23 cm (AOR = 4.939, 95% CI 2.330–10.469), no use of contraceptives (AOR = 4.935, 95% CI 2.207–11.032), and no iron supplementation use (AOR = 3.588, 95% CI 1.794–7.175) were significantly associated with an increased risk of anemia.

**Conclusion:**

According to the WHO classification, anemia in this study was found to be a moderate public health issue. High dietary diversity, no previous excessive menstrual bleeding, and age were significantly associated with a reduced risk of anemia, whereas a MUAC of <23 cm, no contraceptive use, and no iron supplementation were significantly associated with an increased risk of anemia. Therefore, promoting diverse diets among pregnant women, providing counseling on the benefits of family planning and iron-folic acid supplements, and improving women’s education and empowerment are essential.

## Introduction

Anemia is a condition characterized by lower-than-normal hemoglobin (Hb) levels in the body, which reduces red blood cells’ capacity to carry oxygen to tissues. The World Health Organization (WHO) defines anemia based on age, sex, and pregnancy status; for children aged 6 months to 5 years, anemia is defined as an Hb level below 11.5 g/dL; for adult men, it is defined as an Hb level below 13 g/dL; and for non-pregnant women, it is defined as an Hb level below 11 g/dL (1).

This condition affects 41.8% of pregnant women worldwide, with Africa having the highest prevalence (57.1%), accounting for 17.2 million cases (1). Anemia can be caused by multiple factors, such as nutritional (vitamin and nutritional deficiencies) or non-nutritional (infectious) factors (2, 3). However, anemia caused by iron deficiency is one of the top 10 global burdens (4). Approximately half of anemia cases worldwide are caused by iron deficiency (2). Anemia caused by parasitic infections, such as malaria, can reach from 4.5 to 42% (5, 6). Anemia during pregnancy is caused by nutritional deficiencies in iron, vitamin B12, and folate, and parasitic diseases such as hookworm infection and malaria in developing countries such as Ethiopia. In the community, the virtual contribution of each of these factors to anemia varies significantly (5).

Patients with anemia have similar clinical symptoms regardless of the cause. Some of the most common complaints are fatigue, shortness of breath, dizziness, and headaches. During physical examination, the patient might have had pale skin, nails, lips, and palms of the hands. In areas where automated analysis is unavailable, examining a stained blood smear under a microscope for red blood cell morphology is helpful for diagnosing anemia (7).

At the population level, hemoglobin concentration is the most reliable indicator of anemia. Measurement of Hb concentration is a simple and inexpensive procedure frequently used as a proxy indicator of iron deficiency (8). Hb concentration is the most used indicator of anemia at both individual and population levels and is used for screening and assessing intervention programs’ effectiveness. Hemoglobin concentration is typically measured in clinical laboratories using automated hematology analyzers, which are highly reliable and accurate but expensive and immobile in the field (1). Capillary and venous Hb concentrations measured with Hemocue Hb301 showed poor agreement compared to venous Hb measured with automated hematology analyzers, resulting in significantly different anemia prevalences (9).

Anemia in pregnant women has serious consequences for their health, social development, and economic development (2). Anemia is also thought to be a sign of poor nutrition and health. The most dramatic health consequences of anemia, the increased risk of maternal and child mortality due to severe anemia, have been well documented (6).

Furthermore, anemia in pregnant women can lead to various adverse outcomes for both the mother and the newborn. These adverse outcomes include reduced exercise tolerance, an increased risk of puerperal infection, postpartum hemorrhage, pregnancy-induced hypertension, placenta previa, cardiac failure, fetal anemia, low birth weight, intrauterine growth restriction, preterm delivery, and perinatal death (10).

Because anemia is multifactorial, its treatment often requires a multifaceted approach. To effectively combat anemia, contributing factors must be identified and addressed (11). In settings where iron deficiency is the most common cause, additional iron is typically provided to vulnerable groups, particularly pregnant women and young children, through iron supplements. Food-based strategies for increasing iron intake, such as food fortification and dietary diversification, are important long-term strategies for preventing iron deficiency anemia in the general population. Approaches that combine iron interventions with other measures are required in settings where iron deficiency is not the only cause of anemia. Other causes of anemia should be addressed through strategies integrated into the primary healthcare system and existing programs (1).

The presence of anemia as a public health problem necessitates the study of factors associated with anemia to improve the outcomes of current strategies and interventions. Pregnancy covers approximately one-third of the first window of opportunity (1,000 days of life). Because fetal iron requirements take precedence over maternal needs and storage, the continuous assessment of the prevalence of anemia among pregnant women is important to take measures to address its public health problem and to improve intervention to tackle its intergenerational cycle. Therefore, this study aimed to assess the prevalence of anemia and its associated factors, including women’s minimum dietary diversity and the mother’s nutritional status, which might be an important step in decreasing the prevalence of anemia among pregnant women and preventing related complications.

## Materials and methods

### Study design and period

A facility-based cross-sectional study assessed the magnitude of anemia and its associated factors among pregnant women attending antenatal care in public health facilities in Shashemene Town. This study was conducted between 2 April and 28 April 2022.

### Study area

The study was conducted in Shashemene Town, West Arsi zone, Oromia Regional State, Ethiopia, which is located 250 km south of the capital city on the main road from Addis Ababa to Hawassa. It has a latitude of 7°12′ north and a longitude of 38° 36′ east.^1^ The town has 10 kebeles. According to Shashemene Town’s health office statistics for 2022, the projected total population of Shashemene Town was approximately 293,035. Of these, 143,587 were men, and 149,448 were women. Six governmental health facilities (four health centers and two hospitals) provided routine antenatal care services to the community.

### Source population

The source population for the study included all pregnant women who visited governmental health facilities in Shashemene Town.

### Study population

The study population included all pregnant women who visited governmental health facilities during the study period and met the inclusion criteria.

All pregnant women who attended governmental health facilities and provided written informed consent during the study were included.

*Exclusion criteria*: pregnant women:

- Those who had the treatment of anemia and will have a blood transfusion
- Those who had received medication for helminthiasis for the last 3 weeks within the data collection period
- Those who were seriously ill and medically disabled
- Those who had sickle cell anemia or thalassemia

### Sample size determination

#### Sample size determination for the magnitude of anemia in pregnant women

The required sample size for the first specific objective of this study was calculated using the single population proportion formula, based on the 37.8% prevalence of anemia among pregnant women attending the antenatal care (ANC) unit at Najo General Hospital, Northwest Ethiopia, as reported in a study published in October 2020 (12).

The calculated sample size for the first specific objective was:


$$n=\frac{{\left(Z_{a/2}\right)}^{2}p\left(1-p\right)}{d^{2}}$$

where *n* = Minimum sample size; Z = Normal deviant at the portion of the 95% confidence interval two-tailed test = 1.96; p = Prevalence of anemia; d = margin of error acceptable is taken as 5% = 0.05.


$$n=\frac{{\left(1.96\right)}^{2}\left(0.378\right)\left(1-0.378\right)}{{\left(0.05\right)}^{2}}=\frac{0.903221}{0.0025}=361$$

Although the initial sample size was calculated to be 361, it was adjusted to account for a 10% non-response rate, resulting in an increased sample size to ensure adequate data collection.


$$\mathrm{Non}-\mathrm{response rate}=361^{*}10\%=361^{*}10/100=36.1\approx 37$$

$$n_{f}=361+37=398$$

Therefore, the total sample size was **398** participants.

#### Sample size determination for factors associated with anemia in pregnant women

The sample size for the second objective, which was the factor associated with anemia in pregnant women, was calculated based on the double population proportion formula and on the following assumptions: type one error of 5%, power of 80%, and the ratio of exposed to non-exposed 1:1 by taking the odds ratio and percentage of outcome in the unexposed group from previous studies. The above assumptions were substituted into the EPI Info version 7 software Statcalc programs, as summarized in the Table 1.

**Table 1:** Calculation of the second objective sample size based on significant variables of the previous study using EPI-Info 7 statistical software with a power of 80 and 95% CI.

Variables	CI (%)	OR	Ratio	Power (%)	Percent (%) of outcome in the unexposed group	Total sample size	Reference
*Excess menstrual bleeding*	95	9.8	1:01	80	35.69	38	(15)
No (unexposed)							
Yes (exposed)							
*The presence of malaria*	95	0.2	1:01	80	22.1	154	(16)
No (unexposed)							
Yes (exposed)							
*The presence of IPI*	95	3.8	1:01	80	6.28	210	(17)
No (unexposed)							
Yes (exposed)							
*Contraceptive use*	95	2.6	1:01	80	38.1	162	(18)
No (unexposed)							
Yes (exposed)							

The sample sizes identified from the above two objectives were 38, 154, 210, 162, and 361. Since the sample size obtained using the single population proportion formula is the largest of all, with the addition of 10% non-response, the final sample size was **398**.

### Sampling procedure

**Figure 1:**
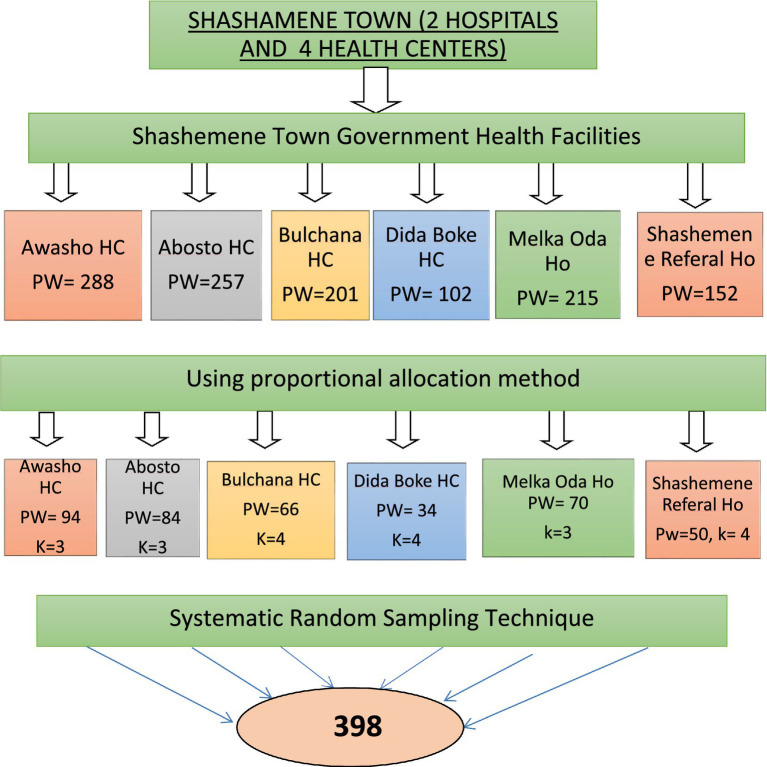
Schematic diagram of sampling procedure of a study on anemia and associated factors among pregnant women attending ANC in government health facilities Shashemene town, Oromia, Ethiopia, 2022. PW, pregnant women; HC, Health Center; Ho, Hospital.

All six governmental health facilities (Awasho Health Center, Abosto Health Center, Bulchana Health Center, Dida Boke Health Center, Melka Oda General Hospital, and Shashemene Referral Hospital) in Shashemene Town were included in this study. The number of study participants was allocated proportionally to the average number of pregnant women who could attend the ANC during the data collection, which was estimated from 1 month’s data of the last year of the same period antenatal care users taken from the ANC registration books of each health facility. Therefore, the sample size of each health facility was calculated by multiplying the average number of pregnant women attending ANC in each health facility per month by the sample size (*n* = 398), divided by the total number of pregnant women attending antenatal care units per month at all health facilities. To select study participants from each facility, the antenatal care registration book containing the list of pregnant women was sought with the reference of 3 months as the sampling frame (Figure 1).

### Study variables

#### Dependent variable

##### Anemia

###### Independent variables

- *Socioeconomic and demographic factors*: age, religion, residence, mother’s education level, ethnicity, family size, and household income.
- *Clinically related factors*: current clinical illness, intestinal parasitic infection, history of excess menstrual bleeding, history of malaria, and deworming.
- *Reproductive health-related factors*: history of contraceptive usage, excess menstrual bleeding, history of abortion, gravidity, parity, trimester, ANC visits.
- *Nutritional factors:* iron supplementation, MUAC, and dietary diversity.

###### Operational definition

*Anemia*: pregnant women with Hb values of <11 g/dL will be considered (1).

*Mild anemia*: pregnant women with an Hb value between 10 and 10.9 g/dL.

*Moderate anemia*: pregnant women with an Hb value between 7 and 9.9 g/dL.

*Severe anemia*: Pregnant women with Hb values of <7 g/dL.

*MUAC*: undernourished (MUAC of <23 cm), normal (MUAC ≥23 cm) (1).

*Women’s dietary diversity score* was the sum of 10 food groups consumed over 24 h before data collection (12).

*Low or poor dietary diversity score*: when a pregnant woman consumed less than five food groups among the 10 groups within 24 h before the survey (12).

*High or good dietary diversity score*: when a pregnant woman consumed five or more food groups among the 10 groups within 24 h before the survey (12).

### The data collection tool and procedures

Data were collected using structured questionnaires that were pre-tested through face-to-face interviews. Sociodemographic questionnaires were adapted from different literature, and maternal dietary practice questionnaires were adapted from the FAO 2019 guidelines for pregnant women (12). All data collectors and supervisors were trained for 2 days before the start of data collection. Six trained B.Sc. midwives were used as data collectors to complete the questionnaires. In contrast, two trained B.Sc. laboratory technologists collected 3 mL of venous blood and stool specimens using clean, dry, leak-proof, and wide-mouthed containers after obtaining informed consent from each participant. One senior health officer was assigned as a supervisor to monitor all the ongoing activities. Subsequently, the hemoglobin concentration was determined using an automated Zybio hematological analyzer. One day of intensive training was provided to both data collectors and supervisors before data collection.

### Laboratory test method

The hemoglobin concentration was determined by a complete blood count (CBC) test using a Zybio automated hematology analyzer. This gold standard is accurate and reliable (33). Laboratory technologists performed the analysis at each health facility. ¾ of the lavender collection tubes (3 mL) of venous whole blood specimen collected in K2EDTA/K3 EDTA (ethylene diamine tetra acetic acid) anticoagulant-coated tube was collected aseptically for each participant, and then hemoglobin concentration was performed.

Moreover, 5 g of stool samples were collected from each study participant after orientation had been given to the women on how to collect sufficient amounts of contamination-free stool specimens using clean, wide-mouthed, and labeled stool cups with leak-proof covers possessing sequential numbers offered to the study participants. The fecal specimen was examined within 10–15 min of collection and then subjected to the wet-mount technique. Fresh stool samples of approximately 2 g were placed on a slide with a wooden applicator, emulsified with a drop of physiological saline (0.85%) enclosed with cover, and examined at 10x and 40x microscopic objectives.

### Minimum dietary diversity: women (MDD-W)

The dietary diversity score was calculated using data from the 24-h dietary recall. Each food group the women ate got a score of "1" or, otherwise, a score of “0.” The number of scores was used to obtain the dietary diversity score (12). Regardless of the number of foods consumed by one food group, a score of “1” was given, and therefore, the total score theoretically ranged from 0 to 10. MDD-W was derived using the dietary diversity score. MDD-W indicates whether a woman has eaten five or more food groups out of the 10 designated food groups within the last 24 h.

The MUAC was measured to assess the nutritional status of the pregnant mother using an adult non-stretchable MUAC tape meter on the participant’s relaxed left arm at the midpoint between the olecranon and acromion.

### Data quality assurance

One day of intensive training before the actual work related to research activities was provided by an investigator and an experienced laboratory technologist to data and sample collectors, who focused on interview techniques, ethical issues, rights of the participants, reading through all the questions and understanding them, and ways of decreasing under-reporting, maintaining confidentiality, and laboratory procedures. An interviewer-administered questionnaire was first prepared in English, translated into Afaan Oromo, and re-translated back into English by language experts to ensure consistency. Moreover, 2 weeks before data collection, the structured questionnaire was pre-tested in line with laboratory procedures at the Adje Health Center. Adjacents were made after pre-testing 5% of the total sample size (20 pregnant women) to ensure quality control. After the pre-test, all necessary adjustments were made. The principal investigator and supervisors checked the completeness and clarity of each questionnaire daily. They also monitored the activities of each data collector through random spot-checking of the individual data collection process for a given sample to ensure the reliability of the data. Any error, ambiguity, incompleteness, or any other related problem was addressed the day before the next day’s activities. However, concerning the quality of sample collection and laboratory procedures, the automated machine had the company produce quality control reagents for easy monitoring. However, to further ensure quality, stool samples were collected in a new container with a long expiry date, and all contents of SOP were followed properly from pre- and post-analysis.

### Data processing, analysis, and presentation

All data from the laboratory report forms and questionnaires were checked for completeness, edited, coded, entered in Epi Data version 3.1, and exported to SPSS Version 21 statistical software for analysis. Descriptive analysis was performed to determine the frequency distribution, and bivariate binary logistic regression analysis was used to test for associations between each independent and dependent variable. Independent variables were selected during bivariate binary logistic regression analysis, with a *p*-value of less than 0.25 entering multivariable logistic regression analysis. Hosmer-Lemeshow statistics were used to check the goodness of fit of the model, and the variance inflation factor (VIF) was used to check for multicollinearity. All variables with *p*-values less than 0.05 at a 95% CI with an adjusted odds ratio (AOR) were considered statistically significant. Finally, the results are presented with narration and descriptive statistics, such as tables, graphs, and charts.

## Results

### Sociodemographic characteristics of the study participants

A total of 391 pregnant women participated in this study, with a response rate of 98% (391/398). The mean ± SD age of the study participants was 24.81 ± 5.653 years (range–15-42). The majority of the study participants, 204 (52.2%), were found to be in the age group of 15–24 (16 study participants were under 18 years old), while 32 (8.2%) study participants were aged > = 35 years. Regarding the religion and ethnicity of participants, 248 participants (63.4%) were Muslim, and the majority of participants, 277 (70.8%), were Oromo by ethnicity. Concerning the participants` educational status, 181 participants (46.3%) had attended primary education, and 86 participants (22.0%) had attended secondary education and above. The majority, 235 participants (60.1%), were housewives. Of the total participants, 337 participants (86.2%) lived in urban areas. Regarding participants’ monthly income, 208 of them (53.2%) earned less than or equal to 1,500 birr per month, with a median of 1500.00 Ethiopian birr (Table 2).

**Table 2:** Sociodemographic characteristics of pregnant women in Shashemene Town, Oromia regional state, Ethiopia 2022 (*n* = 391).

Variables		Frequency	Percentage
Age (years)	15–24	204	52.2
	25–34	155	39.6
	> = 35	32	8.2
Religion	Muslim	248	63.4
	Orthodox	97	24.8
	Protestant	37	9.5
	Waqefata	7	1.8
	Others*	2	0.5
Ethnicity	Oromo	277	70.8
	Amhara	71	18.2
	Gurage	23	5.9
	Others**	20	5.1
Occupation	Housewife	235	60.1
	Farmer	40	10.2
	Merchant	74	18.9
	Student	29	7.4
	Others***	13	3.3
Residence	Rural	54	13.8
	Urban	337	86.2
Education status	Cannot read and write	124	31.7
	Primary	181	46.3
	Secondary and above	86	22.0
Monthly income (ETB)	<=1,500	208	53.2
	1,501–2,500	97	24.8
	> = 2,501	86	22.0

Others*, catholic; others**, Wolayta, Tigre, Silte, and Hadiya; others***, government employee and daily laborer.

### Reproductive health and other related factors

When we examined the reproductive health characteristics of the study participants, more than half of the 210 (53.7%) respondents visited ANC during the second trimester (13–27 weeks) of their gestational age. Regarding gravidity, 240 (61.4%) were multigravida and 178 (45.5%) were multipara. Concerning the birth interval, 155 (39.6%) respondents gave their last birth within 2 years. Most pregnant women who attended antenatal care twice were 175 (44.8%), and those with a family size greater than or equal to five were 235 (60.1%). Of the total respondents, 279 (71.4%) used contraceptives, and 244 (62.4%) did not have a history of excessive menstrual bleeding before pregnancy. Regarding the history of abortion, more than half of the 208 (53.2%) had abortions before the present pregnancy (Table 3).

**Table 3:** Reproductive and other related factors among pregnant women in Shashemene Town, Oromia regional state, Ethiopia 2022 (*n* = 391).

Variables	Categories	Frequency	Percentage
Gestational age	First trimester	16	4.1
	Second trimester	210	53.7
	Third Trimester	165	42.2
Gravidity	Primigravida	151	38.6
	Multigravida	240	61.4
Parity	NulliPara	135	34.5
	PrimiPara	78	19.9
	MultiPara	178	45.5
Birth interval	Never given birth	140	35.8
	1–24 months	155	39.6
	> = 24 months	96	24.6
Family size	< 5	156	39.9
	> = 5	235	60.1
Number of ANC visits	First	147	37.6
	Second	175	44.8
	Third	69	17.6
History of excessive menstrual bleeding	Yes	147	37.6
	No	244	62.4
Previous history of abortion	Yes	208	53.2
	No	183	46.8
History of contraceptive use	Yes	279	71.4
	No	112	28.6

### Clinical-related and other factors

Of the total study participants, approximately 335 (85.7%) respondents had no history of clinical illness at the time of the study. Concerning iron-folic acid supplementation, 238 (60.9%) of the respondents used iron-folic acid supplementation. Regarding the history of having intestinal parasites, more than half of 215 (55.0%) respondents had a history of intestinal parasites. A total of 279 (71.4%) participants had no history of malaria infection before the study, and more than half, 234 (59.8%) of respondents, had no use of deworming in the past 6 months. Of the total study participants, 111 (28.4%) were malnourished with MUAC of <23 cm, and 145 (37.1%) had positive stool examination results for intestinal parasites. Of the total participants, 255 (65.2%) had a high dietary diversity score equal to or greater than five food groups (Table 4).

**Table 4:** Clinica-related and other factors among pregnant women in Shashemene Town, Oromia regional state, Ethiopia 2022 (*n* = 391).

Variables	Categories	Frequency	Percentage
History of clinical illness	Yes	56	14.3
	No	335	85.7
Iron folic acid supplementation	Yes	238	60.9
	No	153	39.1
History of having an intestinal parasite	Yes	215	55.0
	No	176	45.0
History of malaria infection	Yes	112	28.6
	No	279	71.4
History of Deworming	Yes	157	40.2
	No	234	59.8
MUAC measurement	23 cm and above	280	71.6
	Less than 23 cm	111	28.4
Stool examination for intestinal parasite	Positive	145	37.1
	Negative	246	62.9
Dietary diversity score	Low	136	34.8
	High	255	65.2

**Figure 2:**
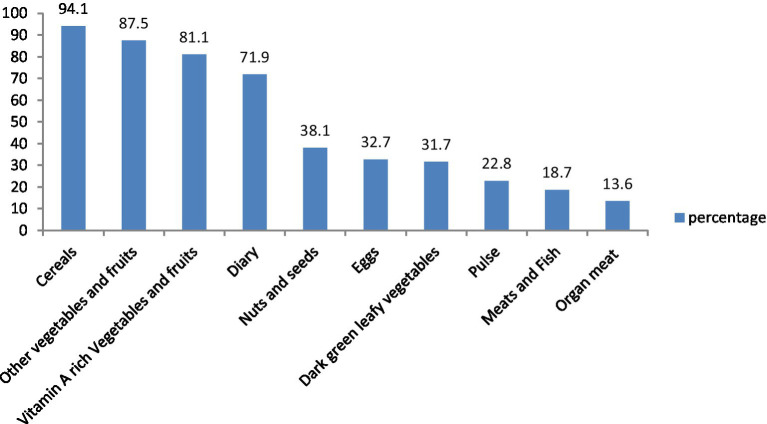
Food groups consumed by pregnant women in Shashemene Town, Ethiopia, 2022.

A large proportion of study participants, 368 (94.1%), had consumed cereal products in the previous 24 h, which was predominant. Vegetables formed an integral part of the main meal for the majority, 342 (87.5%), who consumed other vegetables and fruits. A total of 317 (81.1%) participants consumed vitamin A-rich vegetables and fruits. Concerning animal source food consumption, 281 (71.9%) reported the consumption of milk products (dairy). Nuts and seeds were reported in 149 participants (38.1%). Egg consumption was reported by 148 (37.9%) of the study participants. Dark green leafy vegetables were reported by 124 (31.7%). Plant-based proteins (pulse) were reported by 89 (22.8%) participants. 73 (18.7%) participants minimally consumed meat and fish, and 53 (13.6%) consumed organ meat (iron-rich) (Figure 2).

### Magnitude of anemia among pregnant women

**Figure 3:**
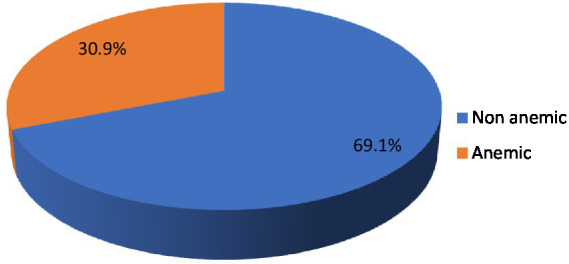
Magnitude of anemia among pregnant women in Shashemene Town, Ethiopia, 2022.

The result of this study shows that the magnitude of anemia among pregnant women was 121 (30.9%) with (95% CI: 26.4, 35.4) with a mean hemoglobin of 11.38 g/dL (SD ± 1.50). The overall magnitudes of mild, moderate, and severe anemia among pregnant women were 73 (18.7%), 42 (10.7%), and 6 (1.5%), respectively. The highest magnitudes of anemia, 89 (73.6%) and 86 (71.1%) were observed among women living in urban areas and those with no use history of deworming in the past 6 months, respectively. The prevalence of anemia among women under 18 years of age was 75% (12 out of 16 pregnant women). Concerning the severity of anemia, the majority, 73 (60.3%), 42 (34.7%), and 6 (5%) of respondents were suffering from mild, moderate, and severe anemia, respectively (Figure 3).

### Factors associated with anemia among pregnant women

In this study, variables associated with the magnitude of anemia in pregnant women were analyzed using bivariate binary logistic regression analysis. Accordingly, variables with a *p*-value of less than 0.25 in the bivariate binary regression analysis were used as candidates for multivariate binary logistic regression analysis. Candidate variables were dietary diversity, nutritional status of MUAC measurement, contraceptive use, history of excessive menstrual bleeding, history of intestinal parasites, education status, residence, family size, history of abortion, parity, gravidity, birth interval, history of malaria, stool examination for intestinal parasite, age, iron supplementation use, history of deworming, and monthly income (Table 5). Variables such as age, dietary diversity score, nutritional status of MUAC measurement, contraceptive use, history of previous excessive menstrual bleeding, and iron supplementation were found to be significantly associated with the odds of anemia (Table 6).

**Table 5:** Bivariate binary logistic regression analysis on factors associated with anemia among pregnant women in Shashemene Town, Oromia regional state, Ethiopia 2022 (*n* = 391).

Variables		Anemia frequency (%)	No anemia frequency (%)	COR (95% CI)	*p*-value
Age	15–24	88 (43.1)	116 (56.9)	**1**	
	25–34	31 (20.0)	124 (80.0)	**0.330 (0 0.204–0.533)**	**0**
	≥ 35	2 (6.3)	30 (93.8)	**0.088 (0.020–0.378)**	**0.001**
Religion	Muslim	72 (29.0)	176 (71.0)	1	
	Orthodox	33 (34.0)	64 (66.0)	1.260 (0.763–2.081)	0.366
	Protestant	16 (43.2)	21 (56.8)	1.862 (0.919–3.772)	0.84
	Others***	0 (0.0)	9 (100)	0	0.999
Occupation	Housewife	75 (31.9)	160 (68.1)	1	
	Farmer	11 (27.5)	29 (72.5)	0.809 (0.384–1.707)	0.578
	Merchant	23 (31.1)	51 (68.9)	0.962 (0.548–1.690)	0.893
	Student	7 (24.1)	22 (75.9)	0.679 (0.278–1.659)	0.395
	Others**	4 (38.5)	8 (61.5)	1.333 (0.422–4.213)	0.624
Education status	Cannot read and write	45 (36.3)	79 (63.7)	**1**	
	Primary	59 (32.6)	122 (67.4)	**0.849 (0.525–1.372)**	0.504
	Secondary and above	17 (19.8)	69 (80.2)	**0.433 (0.2270.824)**	**0.011**
Ethnicity	Oromo	83 (30.0)	194 (70.0)	1	
	Amhara	25 (35.2)	46 (64.8)	1.270 (0.732–2.203)	0.394
	Gurage	8 (34.8)	15 (65.2)	1.247 (0.509–3.053)	0.63
	Others*	5 (25.0)	15 (75.0)	0.779 (0.274–2.214)	0.639
Monthly income (ETB)	<=1,500	76 (36.5)	132 (63.5)	**1**	
	1,501–2,500	30 (30.9)	67 (69.1)	**0.778 (0.465–1.301)**	0.338
	> = 2,501	15 (17.4)	71 (82.6)	**0.367 (0.197–0.685)**	**0.002**
Residence	Rural	32 (59.3)	22 (40.4)	**1**	
	Urban	89 (26.4)	248 (73.6)	**0.247 (0.136–0.447)**	**0**
Gestational age	First trimester	9 (56.3)	7 (43.8)	1	
	Second trimester	74 (35.2)	136 (64.8)	0.423 (0 0.151–1.182)	0.271
	Third Trimester	38 (23.0)	127 (77.0)	0.233 (0.081–0.666)	0.411
Gravidity	Primigravida	62 (41.1)	89 (58.9)	**1**	
	Multigravida	59 (24.6)	181 (75.4)	**0.468 (0.302–0.725)**	**0.001**
	Nullipara	58 (43.0)	77 (57.0)	**1**	
Parity	Primipara	23 (29.5)	55 (70.5)	**0.555 (0 0.306–1.006)**	**0.052**
	MultiPara	40 (22.5)	138 (77.5)	**0.385 (0.236–0.628)**	**0**
Birth interval	never given birth	57 (40.7)	83 (59.3)	**1**	
	1-24 months ago	47 (30.3)	108 (69.7)	**0.634 (0.392–1.025)**	**0.063**
	> = 24 months ago	17 (17.7)	79 (82.3)	**0.313 (0.168–0.584)**	**0**
Family Size	< 5	58 (37.2)	98 (62.8)	**1**	
	≥ 5	63 (26.8)	172 (73.2)	**0.619 (0 0.401–0.956)**	**0.03**
Number of ANC visits	First	84 (57.1)	63 (42.9)	1	
	Second	37 (21.1)	138 (78.9)	0.201 (0.123–0.328)	0.996
	Third	0 (0.0)	69 (100)	0	
History of excessive menstrual	Yes	75 (51.0)	72 (49.0)	**1**	
bleeding	No	46 (18.9)	198 (81.1)	**0.223 (0 0.141–0.352)**	**0**
History of Abortion	Yes	76 (36.5)	132 (63.5)	**1**	
	No	45 (24.6)	138 (75.4)	**0.566 (0.365–0.879)**	**0.011**
History of contraceptive use	Yes	77 (27.6)	202 (72.4)	**1**	
	No	44 (39.3)	68 (60.7)	**1.697 (1.070–2.692)**	**0.025**
History of clinical illness	Yes	32 (57.1)	24 (42.9)		
	No	89 (26.6)	246 (73.4)	0.271 (0 0.152–0.486)	0.285
History of having an intestinal parasite	Yes	83 (38.6)	132 (61.4)	**1**	
	No	38 (21.6)	138 (78.4)	**0.438 (0.279–0.688)**	**0**
Iron folic acid supplementation	Yes	36 (15.1)	202 (84.9)	**1**	
	No	85 (55.6)	68 (44.4)	**7.014 (4.354–11.300)**	**0**
History of deworming	Yes	35 (22.3)	122 (77.7)	**1**	
	No	86 (36.8)	148 (63.2)	**2.025 (1.278–3.209)**	**0.003**
History of malaria infection	Yes	41 (36.6)	71 (63.4)	**1**	
	No	80 (28.7)	199 (71.3)	**0.696 (0.438–1.107)**	**0.126**
Dietary diversity score	Low	78 (57.4)	58 (42.6)	**1**	
	High	43 (16.9)	212 (83.1)	**0.151 (0 0.094–0.242)**	**0**
Stool examination for intestinal parasite	Positive	75 (51.7)	70 (48.3)	**1**	
	Negative	46 (18.7)	200 (81.3)	**0.215 (0.136–0.339)**	**0**
MUAC measurement	23 cm and above	50 (17.9)	230 (82.1)	**1**	
	Less than 23 cm	71 (64.0)	40 (36.0)	**8.165 (4.984–13.376)**	**0**

Bold numerical values indicate candidates for multivariate binary logistic regression analysis. CI, confidence interval; COR, crude odds ratio; AOR, adjusted odds ratio; 1: Reference, Others*, Wolayta, Tigre, Silte, and Hadiya; others**, government employee and daily laborer); others***, Catholic, Waqefata.

**Table 6:** Factors associated with anemia among pregnant women in Shashemene Town, Oromia region, Ethiopia, 2022 (*n* = 391).

Variables		Anemia Frequency (%)	No anemia Frequency (%)	COR (95%CI)	AOR (95%CI)	*p*-value
Dietary diversity score	Low	78 (57.4)	58 (42.6)	1	1	
	High	43 (16.9)	212 (83.1)	0.151 (0.094–0.242)	0.217 (0.105–0.451)***	<0.001
MUAC measurement	23 cm and above	50 (17.9)	230 (82.1)	1	1	
	Less than 23 cm	71 (64.0)	40 (36.0)	8.165 (4.984–13.376)	4.939 (2.330–10.469)***	<0.001
History of contraceptive use	Yes	77 (27.6)	202 (72.4)	1	1	
	No	44 (39.3)	68 (60.7)	1.697 (1.070–2.692)	4.935 (2.207–11.032)***	<0.001
History of excessive menstrual bleeding	Yes	75 (51.0)	72 (49.0)	1	1	
	No	46 (18.9)	198 (81.1)	0.223 (0.141–0.352)	0.162 (0.076–0.345)***	<0.001
Iron folic acid supplementation	Yes	36 (15.1)	202 (84.9)	1	1	
	No	85 (55.6)	68 (44.4)	7.014 (4.354–11.300)	3.588 (1.794–7.175)***	<0.001
Age	15–24	88 (43.1)	116 (56.9)	1	1	
	25–34	31 (20.0)	124 (80.0)	0.330 (0.204–0.533)	0.391 (0.173–0.883)*	0.024
	≥ 35	2 (6.3)	30 (93.8)	0.088 (0.020–0.378)	0.068 (0.011–0.444)**	0.005

1 = Reference; * *p*-value of < 0.05, ** *p*-value of < 0.01, ***p-value of < 0.001; COR, crude odds ratio; AOR, adjusted odds ratio; CI, confidence interval; gestational age, parity, gravidity, birth interval, education status, residence, family size, stool examination for intestinal parasites, history of having intestinal parasites, history of abortion, history of malaria, stool examination for intestinal parasites, iron supplementation use, history of deworming, and monthly income factors were found to be confounders.

Dietary diversity was found to be significantly associated with anemia among pregnant women, with a high dietary diversity score of 78.3% (AOR = 0.217, 95% CI 0.105–0.451) and lesser odds of developing anemia compared to pregnant women with a low dietary diversity score.

Nutritional status was significantly associated with anemia during pregnancy; pregnant women with MUAC less than 23 cm had 4.939 (AOR = 4.939, 95% CI: 2.330–10.469) times higher odds of developing anemia compared to pregnant women with MUAC greater or equal to 23 cm.

History of contraceptive use was the other predictive variable showing a significant association with anemia during pregnancy, and the odds of having anemia among pregnant women who had no history of contraceptive use before this pregnancy were 4.935 (AOR = 4.935, 95% CI: 2.207–11.032) times higher odds of developing anemia compared to pregnant women who had a history of contraceptive use before pregnancy.

Excessive menstrual bleeding was significantly associated with anemia in pregnant women. Women who had previous excessive menstrual bleeding before this pregnancy had 16.2% (AOR = 0.162, 95% CI: 0.076–0.345) higher odds of developing anemia compared to pregnant women who had no previous excessive menstrual bleeding.

Regarding iron supplementation, pregnant women who did not take iron supplementation during pregnancy were 3.5 (AOR = 3.588, 95% CI: 1.794–7.175) times more likely to develop anemia than pregnant women who took iron supplementation.

Another factor, the age of pregnant women, showed a significant association with anemia during pregnancy; the odds of having anemia among pregnant women aged 25–34 years were 60.9% (AOR = 0.391, 95% CI: 0.173–0.883), and those aged ≥35 years were 6.8% (AOR = 0.068, 95% CI: 0.011–0.444) less likely to develop anemia than pregnant women aged 15–24 years.

## Discussion

Anemia is a global health issue affecting both developing and developed countries, with serious implications for human health and social and economic development (2). Anemia was found to be a moderate public health problem, with 30.9% of pregnant women suffering from anemia in the study area, according to the WHO classification (2).

The prevalence of anemia in pregnant women in the study area was lower than that in studies conducted in Bathinda, India (81.8%), Malaysia (57.4%), Northern Ghana (50.8%), Gonja District of Ghana (55.8%), Goba, Bale Zone (46.2%), Nejo Hospital (37.8%), and Wolayita Soddo, Town (39.94%) (13–19). However, compared to studies conducted in Turkey (27.8%), Kampala, Uganda (25.8%), Millennium Medical College Addis Ababa (11.6%), Adigrat (7.9%), Debre Berhan (9.7%), West Gojjam Zone (10.6%), Hossana (24.2%), and Adama (28.1%) (20–27). A possible reason for this inconsistency might be disparities in socioeconomic position, altitude differences, and dietary habits of pregnant women.

Regarding the degree of anemia among pregnant women, this study found that approximately 60.3, 34.7, and 5% of study participants had mild, moderate, and severe anemia, respectively. A similar study with the highest mild type, lower moderate type, and lowest severe type of anemia in pregnant women was observed in Debre Berhan, in which the majority of the cases had mild anemia (64.29%), moderate anemia (32.1%), and severe anemia (3.57%). However, this study is quite different from a study done in Wolayita Soddo Town, in which the majority (60%) of the study participants were moderately anemic, 30.34% were mildly, and 9.66% were severely anemic (13, 22). The similarities and discrepancies in the study results could be attributed to geographic location, food habits, and socioeconomic position.

Predictive factors, such as history of contraceptive use, history of excessive menstrual bleeding, age, iron supplementation, nutritional status of MUAC measurement, and dietary diversity score, were significantly associated with anemia among pregnant women. This study showed a positive association with a history of contraceptive use, which means pregnant women who had no history of contraceptive use before were 4.935 (AOR = 4.935, 95%CI 2.207–11.032) times more likely to be anemic during pregnancy. The result is congruent with that of a study conducted in Hawassa and Wolayita Sodo Town, which states that the odds of being anemic were significantly higher among women not using contraception than among those using contraception (13, 28). The same condition could be caused by the use of contraceptive techniques to prevent pregnancy and childbirth problems, which could eventually decrease the prevalence of anemia due to recurring blood loss.

Another factor associated with anemia in pregnant women is a history of excessive menstrual bleeding. Pregnant women with a history of excessive menstrual bleeding before pregnancy were also significantly associated with anemia. Accordingly, pregnant women who had previous excessive menstrual bleeding had 16.2% (AOR = 0.162, 95% CI: 0.076–0.345) higher odds of developing anemia compared to pregnant women who had no previous excessive menstrual bleeding. The results of this study are comparable to those of studies conducted in Wolayita Sodo Town and Horo Guduru (13, 28). This resemblance could be related to the fact that iron levels can decrease because of blood loss during menstruation.

This study showed that the nutritional status of pregnant women is associated with anemia during pregnancy. Pregnant women with an MUAC of <23 cm during pregnancy were 3.5 (AOR = 3.588, 95% CI 1.794–7.175) times more likely to develop anemia. This result is congruent with a study conducted in Adare, which was 4.939 (AOR = 4.939, 95% CI 2.330–10.469) times more likely to be anemic compared to non-malnourished women. This result is consistent with that of a study conducted in Horo Guduru, Adama Town, and Jigjiga, East Ethiopia (25, 28, 29). These similarities could be related to the fact that anemia can be caused by malnutrition, which lacks micronutrients.

Pregnant women who were not taking iron supplementation during pregnancy were 3.5 (AOR = 3.588, 95% CI: 1.794–7.175) times more likely to develop anemia. The result is congruent with that of a study conducted in Adare General Hospital (30). The reason for this might be that lack of iron supplementation is among the most significant risk factors for developing anemia during pregnancy. This is likely because the requirement for iron increases for pregnant women compared with non-pregnant women, and if they do not receive iron supplementation, the iron they ingest from food sources is not adequate to meet their needs.

Pregnant women aged 25–34 years were 60.9% (AOD = 0.391, 95% CI: 0.173–0.883), and those aged > = 35 years were 6.8% (AOD = 0.068, 95% CI: 0.011–0.444) less likely to develop anemia compared to pregnant women who were aged 15–24 years. The result is congruent with that of a study conducted in Wolayita Sodo Town (13, 31). This could be due to anemia in young pregnant women, which has been attributed to the fact that they are still growing and need additional iron and folic acid to meet their own nutritional needs and those of the developing fetus during gravidity. Older pregnant women have already built up a reserve of iron in their bodies over the years, which can help prevent the development of anemia during pregnancy. In addition, this could be due to better self-dependency, educational level, and health decision-making in taking good care during pregnancy in older women than in younger pregnant women.

The present study also identified that pregnant women with a high dietary diversity score of 78.3% (AOR = 0.217, 95% CI 0.105–0.451) were less likely to develop anemia than pregnant women with a low dietary diversity score. This finding was consistent with those of studies conducted in Northern Ghana, Horo Guduru, Hossana, and Mekelle (23, 28, 32). This could be because pregnancy is a phase that requires physiologically high nutrition, and nutrition requirements increase dramatically not only to fuel pregnant women but also the growing fetus. Inadequate dietary iron intake and insufficient iron are found in the food they eat. Consequently, the iron levels decreased.

Generally, this study’s findings can have significant implications for national nutrition strategies and for promoting essential nutrition actions, especially for improving women’s nutritional status during pregnancy.

Furthermore, the findings indicate the factors that may contribute to anemia during pregnancy, allowing policymakers and healthcare providers to plan and implement contextually relevant interventions to reduce anemia and its associated negative consequences. The findings also provide a basis for large-scale, quantitative, and qualitative studies in different contexts in Shashemene Town to estimate anemia prevalence, identify important determinants, and investigate detailed contextual, structural, and personal-level explanatory factors.

### Strength of the study

This study provides valuable information regarding anemia during pregnancy and its risk factors in Shashemene Town. The study’s findings are relevant for informing public health interventions to stakeholders, policymakers, and the Ministry of Health to reduce the risk of anemia.

Dietary diversity and anthropometric parameters such as MUAC were assessed. Blood and stool samples were collected from all study participants. Comprehensive statistical analyses were performed to address multiple variables related to anemia.

### Limitation of the study

The cross-sectional study design that we used could not infer causality.

This facility-based cross-sectional study exclusively included pregnant women who had antenatal care followups, which might undermine the generalization of the study results to all pregnant women left in the community or who did not attend antenatal care followups.

This study also did not detect the malaria parasite in the blood and conducted a multiple-pass 24-h technique to estimate the usual food intake of pregnant women because of financial constraints to do so.

## Conclusion

Anemia in pregnant women was found to be a moderate public health problem in the study location (30.9%) according to the WHO cut-off values for public health significance (2). Anemia in pregnant women was found to be significantly associated with high dietary diversity, nutritional measurement of an MUAC of <23 cm, no contraceptive use, no previous excessive menstrual bleeding, no iron supplementation, and the age of pregnant women. High dietary diversity, no previous excessive menstrual bleeding, and age were significantly associated with a reduced risk of anemia. Factors such as an MUAC of <23 cm, no contraceptive use, and no use of iron supplementation were significantly associated with an increased risk of anemia. Nutritional assessment and education or counseling on the consumption of iron-rich foods and iron-folic acid supplements, as well as counseling on the benefits of utilizing contraception and employment opportunities for young people, should be enhanced to improve women’s educational level and empowerment.

## Recommendations

At the community level:

- Health extension workers should promote nutrition education to improve women’s nutritional knowledge when visiting pregnant mothers at home and during different contact points such as community health days, pregnant forums, one-five networks, and women’s development army meetings.

At the Health Facilities level:

- Health workers should provide nutrition education and counseling on each antenatal visit on a variety of foods, focus on locally available foods, frequency of foods, hygiene, folic acid supplementation, and prevention of anemia to promote maternal health and good pregnancy outcomes.
- Nutritional status assessment
- Information, education, and communication activities regarding family planning services and encouraging and broadening the activities of health workers.

Shashemene Town Health Office, Educational Office, and Agriculture and Rural Development Office:

- The health office and the regional health bureau and Ministry of Health (MOH) should jointly conduct further community-based studies in the general population to ensure further representativeness.
- The health office should enforce community linkages and MCH to benefit women with better nutritional knowledge.
- The educational office should provide public awareness regarding girls’ education and empowerment.
- The agricultural office enables them to produce foods locally for both consumption and commercial purposes so that they are able to meet both nutritional and financial demands with minimal compromises.

To the researchers:

- Researchers should perform laboratory tests to detect malaria parasites in the blood and assess the association between malaria parasites and anemia.
- To reduce recall bias, it would be better to use the multiple-pass 24-h technique to estimate the usual food intake of pregnant women.
- It would be better if the study were case-controlled and supplemented with a qualitative study.

## Data Availability

The raw data supporting the conclusions of this article will be made available by the authors, without undue reservation.

## Footnotes

^1^ http://mui.gov.et/web/shashmene/home
